# Cervical Cancer Classification From Pap Smear Images Using Deep Convolutional Neural Network Models

**DOI:** 10.1007/s12539-023-00589-5

**Published:** 2023-11-14

**Authors:** Sher Lyn Tan, Ganeshsree Selvachandran, Weiping Ding, Raveendran Paramesran, Ketan Kotecha

**Affiliations:** 1 Institute of Actuarial Science and Data Analytics, UCSI University, Jalan Menara Gading, Cheras, 56000 Kuala Lumpur, Malaysia; 2 School of Business, Monash University Malaysia, Jalan Lagoon Selatan, Bandar Sunway, 47500 Subang Jaya, Malaysia; 3 Symbiosis Institute of Technology, Symbiosis International University, Pune, 412115 Maharashtra India; 4 School of Information Science and Technology, Nantong University, Nantong, 226019 China; 5 School of Information Technology, Monash University Malaysia, Bandar Sunway, 47500 Subang Jaya, Malaysia; 6 Department of Electrical Engineering, Faculty of Engineering, University of Malaya, Kuala Lumpur, Malaysia; 7 Symbiosis Centre for Applied Artificial Intelligence, Symbiosis International (Deemed University), Symbiosis Institute of Technology, Pune, 412115 India

**Keywords:** Cervical cancer classification, Cervical cancer detection, Pap smear images, Convolutional neural network, Deep learning, Medical image processing

## Abstract

As one of the most common female cancers, cervical cancer often develops years after a prolonged and reversible pre-cancerous stage. Traditional classification algorithms used for detection of cervical cancer often require cell segmentation and feature extraction techniques, while convolutional neural network (CNN) models demand a large dataset to mitigate over-fitting and poor generalization problems. To this end, this study aims to develop deep learning models for automated cervical cancer detection that do not rely on segmentation methods or custom features. Due to limited data availability, transfer learning was employed with pre-trained CNN models to directly operate on Pap smear images for a seven-class classification task. Thorough evaluation and comparison of 13 pre-trained deep CNN models were performed using the publicly available Herlev dataset and the Keras package in Google Collaboratory. In terms of accuracy and performance, DenseNet-201 is the best-performing model. The pre-trained CNN models studied in this paper produced good experimental results and required little computing time.

**Graphical Abstract:**

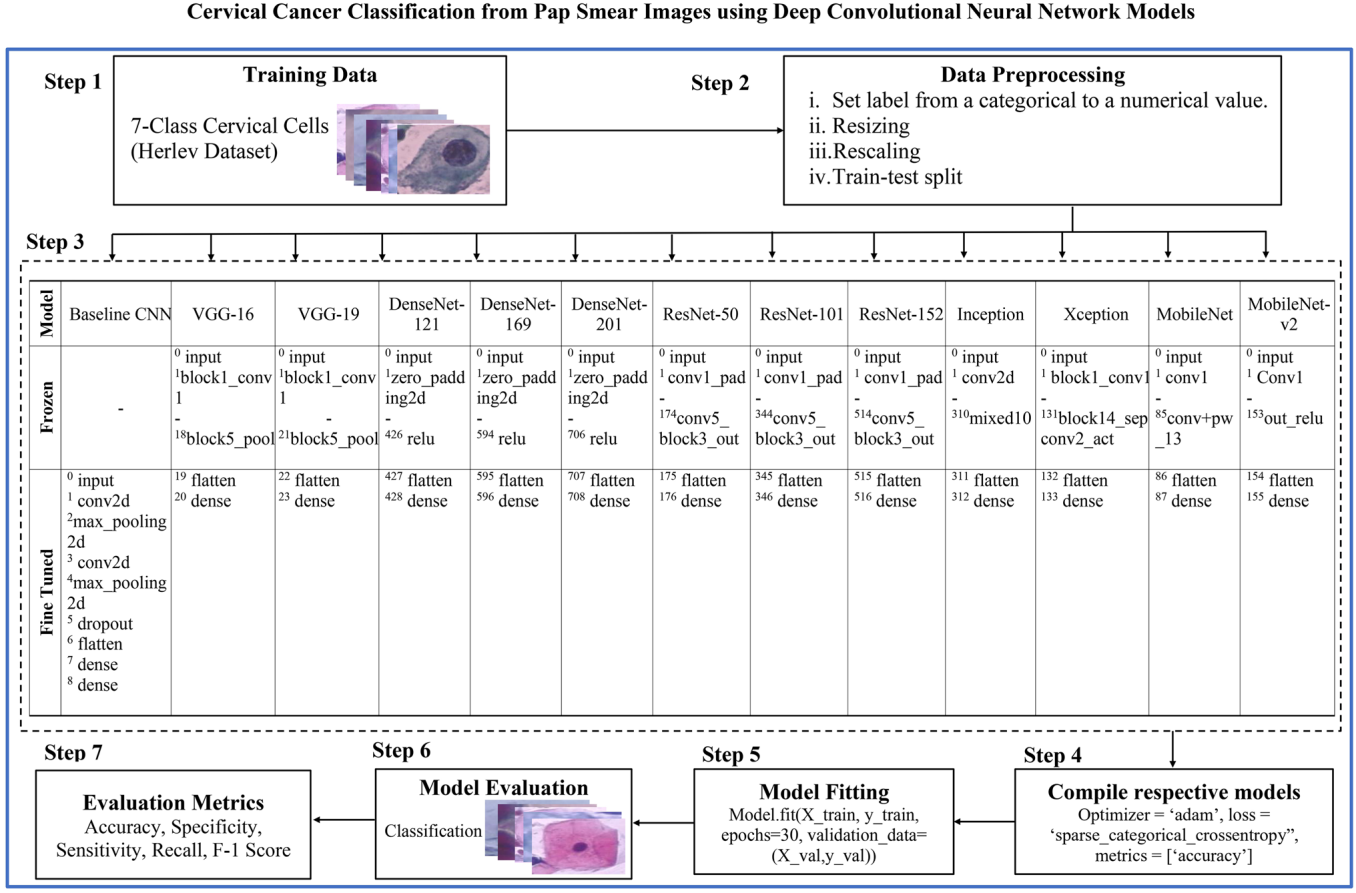

## Introduction

Cervical cancer stands out among women’s cancers as the fourth most common cancer [1]. A healthy cell may take several years to transform from a prolonged and reversible pre-cancerous lesion stage into a malignant cervical cancer [2]. Since cervical cancer is treatable if discovered at an initial pre-cancerous phase and further cancer progression can be averted [3], thus, the timely identification of the disease is the key to lessening the overall burden of the disease on society.

The detection of cervical cancer can be aided by cytology examinations, with the Pap smear test being the most widely recognized and easily accessible screening method. However, examining the Pap smear slides beneath the microscopes is still a challenging, laborious, and manual operation, even with expert cytopathologists due to the requirement of cytopathologists to review numerous micro-images within a single slide for each patient screened and the irregularities were often remained undiscovered because of avoidable human errors [4]. Additionally, the cervical intraepithelial neoplasia is rather tiny, and clumps of cells are overlapped or masked by blood mucus, which complicated the Pap smear images, thus making the procedure highly error-prone [5]. Emerging research suggests that computer-aided techniques can play a significant role in automating cancer diagnosis. Despite positive findings, there are still deficiencies with the current methods for cervical cancer diagnosis and classification that need to be resolved.

First, the authors in [5–8], and [9] have noted that the Pap smear test heavily relies on manual evaluation and analysis of microscope images. This manual examination process has proven to be laborious, expensive, incredibly time-intensive, and highly susceptible to human errors as there are approximately three million cells with different orientations, sizes, and shapes, and many of the cells were overlapping. Second, in contrast to manual assessment, a growing body of research has been dedicated to the advancement of computer-aided diagnostic (CAD) tools that can automatically classify abnormal cervical cells from cytology specimens. Segmentation of cell, feature extraction, and classification are parts of the traditional CAD tools [8]. The primary drawback of CAD systems is that the classification quality is not guaranteed because the extracted features used for classification were often hand-crafted, in contrast to deep learning (DL) which uses medical image inputs directly without manual intervention and does not necessarily require careful design at all stages [10]. On the other hand, machine learning (ML) has recently attracted the interest of academics in studying if these approaches are suitable and robust enough to be employed as an alternative tool for addressing the difficulties in clinical diagnostic problems. Noticeably, convolutional neural network (CNN) in particular, have demonstrated prospects for enhancing the performance of cervical cancer detection and diagnosis [5] with its ability in identifying and learning high-level features and hidden patterns directly on input images.

From the literature review, there are three primary categories into which the current development of cervical cancer detection and classification approaches may be divided: (1) object detection-based approaches, (2) segmentation-based approaches, and (3) end-to-end classification of cervical cells without prior segmentation and feature extraction.

Cervical cancer cell classification using object detection-based approaches has grown popular recently [10], and CNN-based object detection frameworks have been employed in several studies to classify and locate cervical cells as seen in [9–14], and [15]. These studies attempted to automate the diagnosis process by analyzing the image data directly at the image level instead of at the pre-processed cellular level.

Conventional classification approaches typically involve segmentation of cells and cell feature extraction stages for subsequent classification stages [3, 16]. This makes the classification quality dependent on the effectiveness of cell segmentation. Classifiers often faced challenges in identifying overlapping cells with vague cytoplasmic boundaries. Since the nucleus is a dependable source of information for cervical cancer screening, precise segmentation of nuclei and cytoplasm is essential. OTSU and DRLSE algorithms were adopted by Bao et al. [17] to segment the contours of cervical nuclei and William et al. [4] employed the Trainable Weka Segmentation (TWS) toolkit for cell segmentation. In [4], the authors also put in extra efforts to post-process the segmented images with a sequential elimination approach to remove debris that can affect the classification accuracy. Both pre-processing and post-processing of the images are essential steps in the segmentation-based approach.

Both object detection-based and segmentation-based approaches required manual intervention on the image datasets before the images were fed into a classifier. The location and segmentation tasks became difficult for complex data patterns in raw medical images that are associated with overlapping cells and debris. These subsequently inspired the development of end-to-end classification methods that directly operate on raw images and do not necessitate custom features. Additionally, methods based on deep learning approach made a significant advance in this area by providing encouraging accuracy. Many authors used CNNs algorithms such as DenseNet-121, DenseNet-169, AlexNet, VGG-16, VGG-19, ResNet-50, ResNet-101, GoogleNet, Inception-v3 and Xception with transfer learning to perform classification of cervical cells as presented in [6, 18, 19], and [20]. Meanwhile, to utilize the benefits of multiple CNN models for the classification tasks parallelly, Hussai et al. [6] and Manna et al. [19] presented ensemble models to aggregate the top-performing models to develop a more generalized model for the classification.

Nonetheless, most works on the cervical cancer cells classification use the Herlev dataset [21] which is from a public database that consist of seven imbalanced classes of cells. Moreover, there exist private research datasets in addition to public datasets. Regrettably, most researchers are unable to access private databases and are forced to conduct their research using the small-sized public Herlev dataset. To overcome the challenge of limited data, most of the surveyed studies relied on transfer learning techniques, which involves leveraging pre-trained models from one task and adapting them to another task, effectively utilizing existing knowledge and models in the face of data scarcity [6, 18–20].

In this study, a comprehensive end-to-end classification of cervical cancers on the publicly available Herlev dataset without the need for separate feature extraction and segmentation processes was conducted. To compensate for the limited data size and class imbalance problem in the underlying Herlev dataset, CNN models with transfer learning methods were used. A range of CNN networks, namely VGG-16, VGG-19, DenseNet-121, DenseNet-169, DenseNet-201, ResNet-50, ResNet-101, ResNet-152, Inception, Xception, MobileNet, and MobileNet-v2 networks were evaluated on the Herlev dataset. The objectives of this study are: (1) to provide a thorough evaluation and comparison of pre-trained deep CNN models for detecting cervical cancer using publicly available datasets and (2) to examine the effectiveness of CNN models with transfer learning in multi-class classification tasks on imbalanced image datasets.

In contrast to previous studies and reviews that lack clear documentation of essential details like architecture, hyperparameters, and training methodologies of CNN models, this research offers a comprehensive evaluation of existing CNN models for cervical cancer classification, systematically compiling them for easy reference, and setting a benchmark for future research. By filling this gap, the study enables better comparison and benchmarking of different models, promoting transparency and facilitating advancements in the field. The main contributions of the present study are as follows:

(i) This research presents an extensive evaluation of established CNN models using transfer learning approach, specifically tailored for classifying cervical cancer. It is worth noting that this level of comprehensive and detailed analysis has not been previously undertaken, thus making this study distinct and highly valuable.
(ii) In this study, a direct approach was adopted, where Pap smear images were directly processed for automatic detection of cervical carcinomas from the public Herlev dataset, eliminating the need of discrete design at each stage. The compilation of models and the corresponding results were obtained under similar experimental conditions, facilitating easy comparison and accessibility for the research community focusing on cervical cancer classification.
(iii) Unlike previous studies that primarily focused on binary classification, this study specifically addresses the classification of cervical cancer subtypes, providing valuable insights into the performance of various CNN models for handling classification into multiple classes, particularly with imbalanced datasets.

The following sections of this paper are structured in the subsequent manner: insight into prior studies concerning cervical cancer detection is presented in Sect. 2. Section 3 outlines the dataset and research methodology employed. Section 4 presents the computational results, and the findings are covered in Sect. 5. Section 6 provides final remarks, followed by acknowledgments and the reference list.

## Related Works

Deep learning (DL) already outperformed human specialists in the domain of modeling highly complicated connections between inputs and outputs, where the features are not human-understandable [22]. In [23], the authors concluded that there are currently no designated methods for defining the proper deep network parameters and proposed that the existing deep learning models, which were initially designed for other tasks and already achieved excellent performance, could be further enhanced by tuning or refining their model structures for medical images. Litjens et al. [24] highlighted that the utilization of DL techniques for medical image classification and related tasks is a rapidly expanding research field. In this area, CNN is a common deep architecture in this discipline and has demonstrated significant achievements in cell detection, segmentation, classification, localization of regions of interest, and state-of-the-art accuracy [25]. The model’s biggest drawback for building the model from scratch is that it needs a sizable amount of annotated data for training. Not to mention that it takes a lot of computing power and a lengthier training duration. Transfer learning, which applies the trained model that was trained from one task to a new task, offers a solution to all these issues. Furthermore, most of the existing DL studies on Pap smear images either focus primarily on two-class classification, also widely known as binary classification or taking single cell images rather than raw medical images [6]. This section provides an overview of the importance of ML and DL techniques, along with their evolutionary progression in the domain of cervical cancer diagnosis.

### Detection of Cervical Cells Based on Object Detection Approaches

Contrary to classification, detection requires an added location task. The detection network comprises a detection head to search the specified object region. The two most common forms of detection networks are (1) one-stage systems and (2) two-stage systems. One-stage detection networks directly perform location prediction in a single stage without establishing region proposals. Two-stage detection networks first establish region proposals as a pre-detection step, then calibrate the location and perform classification [12].

Elakkiya et al. [15] introduced the Faster Small-Object Detection Neural Networks (FSOD-GAN) for automated identification of cervical spots, achieving a classification accuracy of 99%. In [12], the authors formulated 3cDe-Net, based on a dilated convolution ResNet and multiscale feature fusion through feature pyramid network (FPN), which had achieved superior performance compared to existing approaches with a MAP of 50.4%. This network processes directly at the cervical image level as opposed to the cell level and can identify cells with a variety of sizes and scales.

A new detection network, the DGCA-RCNN model, was presented by Li et al. [5] for detecting abnormal cervical cells from pathology images was proven robust in identifying subtle differences between types of cervical cells. Meanwhile, it was also noted that mAP decreased while IOU was increasing, suggesting that great details were needed for the classifier to learn the complex attributes, and that it is preferable to obtain magnified image patches to enable accurate detection of malignant cells.

Nambu et al. [13] resolved the challenge of classifying cell clusters that overlap by introducing a two-stage CNN algorithm to classify crowded and overlapping cell images. The authors first applied You Only Look Once v4 (YOLOv4) for cell detection then applied ResNeSt to perform the classification task. Moreover, Bai et al. [11] introduced an improved Faster RCNN (CLDNet) model to compensate the problem with manual colposcopy reading by enhancing the lesion attributes with Squeeze-Excitation CNN (SE-CNN) to capture the deep features. Alsalatie et al. [9] presented an ensemble DL model, which applied Faster and enhanced Region-CNN model to locate cervical regions, the CLS-net for feature extraction, followed by an ensemble of two CNN models, whereby the initial model trained and classified cells into normal or abnormal classes, while the second model further trained and classified the abnormal cases into the three classes. The accuracy of the proposed ensemble model demonstrated its superiority over the existing methods in literature where multi-class classification on whole slide images was conducted.

A CNN-based object detection approach was developed by Xiang et al. [10]. They utilized a YOLOv3 base model for detecting cervical cells within whole slide images and incorporated the InceptionV3 base model to enhance classification accuracy. The proposed approach demonstrated effective image-level classification without requiring cell segmentation with high accuracy and sensitivity rate of nearly 100%, but the low specificity at 67.8% and the authors associated these subpar results primarily with the severely unbalanced data distribution.

### Classification of Cervical Cells

Image acquisition, image pre-processing, image segmentation, feature extraction, and classification are typically the key phases in medical image analysis [26]. There have been several classification methods proposed in recent years, and a number of them involved segmentation or the extraction of texture features.

#### Classification of Cervical Cells Based on Segmentation or Feature Extraction

Recently, Bao et al. [17] compared the performance of intelligent cytology system and manual reading by an experienced cytologist. They first segmented the contours of cervical nuclei using OTSU and DRLSE algorithms and then performed classification through VGG-16 and the study achieved equivalent results in terms of sensitivity and specificity relative to manual reading.

In [4], the authors minimized the likelihood of error by automating the procedure with the application of contrast local adaptive histogram equalization to enhance Pap smear image quality, Trainable Weka Segmentation (TWS) classifiers for segmentation of cells, sequential elimination technique for noise reduction, feature selection with simulated annealing with wrapper filter and fuzzy c-means (FCM) for the classification. The selected salient features significantly improved the performance of the FCM algorithm, contributing to a low classification error rate. Apart from that, by combining feature vectors extracted from several CNN architectures to allow the model to capture more potential information and, hence, improve the class, a hybrid deep feature fusion technique was used by Rahaman et al. [7] to develop DeepCervix. The general accuracy of the individual DL models decreased with the expansion of the number of classes, except in the case of the suggested hybrid deep feature fusion technique. Moreover, Alquran et al. [27] devised a novel feature extraction method using their newly introduced Cervical Net structure followed by feature fusion using the Shuffle Net structure and the extracted features were then passed to different ML classifiers. The fusion feature extraction method had varying effects on the performance of classifiers. SVM and Naive Bayes showed improved performance, but RF, KNN, and ANN performed worse.

Park et al. [28] compared a range of ML and DL models for binary cervical cancer classification. They first extracted features by pyradiomics 3.0, then selected significant features using the Lasso model, and fed them into the XGB, SVM, RF, and ResNet-50 models, respectively, to classify images into positive or negative instances. The results indicated that the ResNet-50 algorithm outperformed the non-DL models. In [14], the authors applied progressive resizing for morphological cell feature extraction and employed a pre-trained Conv Net to classify cervical cells into multiple instances. The incorporation of progressive resizing significantly improved the multi-class classification outcomes, resulting in excellent sensitivity, specificity, and Kappa scores for the proposed methodologies. In contrast, Li et al. [29] presented a classifier based on multilayer hidden conditional random fields for assigning labels to cervical cancer stages. Still, their approach required scale-invariant feature transform for extraction of features and feature selection based on Gaussian distribution.

In [30], the authors devised GLCM+Gabor model for feature extraction and used the LeNet-5 model for abstract feature extraction in parallel. The strong features and abstract features were fused and inputted into the SVM classifier. Compared to the CNN-SVM alone, the CNN-SVM with a strong feature showed slight improvement, suggesting that the inclusion of a strong feature could potentially enhance the models’ performance and reliability in detecting positive cells. In [31], the authors addressed the problem of lost domain knowledge and missing features in cervical cell classification by employing artificial feature extraction. These extracted features were combined with InceptionV3. The enhanced InceptionV3 algorithm with artificial features outperformed the classic InceptionV3 network in terms of accuracy. However, the authors emphasized the need for further research and analysis to merge artificial and deep neural network-generated features.

#### End-to-End Classification of Cervical Cells Based on Deep Learning Approaches

To simplify and reduce computational complexity, researchers have utilized neural networks (NN) for fully automated classification, eliminating the need for separate image enhancement, detection, segmentation, and feature extraction steps.

In [32], the authors adopted transfer learning (TL) techniques and formulated PsiNet-TAP model, which adopted an adaptive pruning method based on the product of ${l}_{1}$-norm and output excitation means that can directly perform classification on unprocessed Pap smear images. The proposed pruning approach demonstrated an alternate optimization method that greatly reduced the network size and hence shortened the computational time and improved the performance of classifiers. In both [18] and [20], TL techniques with ResNet-50, DenseNet-121, and DenseNet-169 were used for binary classification to overcome data limitations. In [20], an image processing technique based on an acetowhite mask image was proposed, allowing the model to concentrate on the pertinent region during training. In [18], pre-trained DenseNet was employed to classify lesion levels in cervical images. DenseNet-169 outperformed DenseNet-121 in accuracy and sensitivity, suggesting a positive correlation between network depth and sensitivity. The DenseNet-based classifiers outperformed SVM classifiers trained with custom features. Notably, the DenseNet models analyzed 600 images in less than a minute.

Most studies focused on binary classification of cervical cancer. Only a few, such as [6, 19, 32], explored end-to-end multi-class classification using DL approaches without the need for cell segmentation and feature extraction. In [6], an innovative ensemble classifier was introduced for multi-class classification. The output of six classifiers (VGG-16, VGG-19, Alexnet, ResNet-50, ResNet-101, and GoogleNet) were examined, and the top three models were combined using a voting strategy to create the ensemble classifier. Compared to other models, the proposed ensemble classifier demonstrated significantly higher AUC values, outperforming Alexnet, VGGNet, ResNet, and GoogleNet. It demonstrated robustness by classifying Pap smear images without segmentation techniques. The authors considered it the most generalized model as it integrated three optimized CNN models. In [19], the authors presented an ensemble model with a novel fuzzy rank-based fusion technique to ensemble the top three CNN classifiers, leading to enhanced classification performance. However, they observed that some images with blur or overlapping cells could not be correctly identified, implying the need for pre-processing techniques.

Classification tasks typically require a large volume of quality images with annotation and balance distribution. Zhao et al. [33] innovatively resolved this challenge using a taming transformer (CCG-taming transformers) along with the introduction of new convolutional structures for data augmentation and presented Tokens-to-Token Vision Transformers to perform multi-class classification. The CCG-taming transformers generated images that closely resembled actual cervical cells, serving as an effective training dataset leading to improved classification accuracy.

## Summary

The reviewed literature indicates a rising interest in the application of AI tools for cervical cancer screening. However, the studied algorithms suffer from the following limitations and challenges:

(i) Limited Data Size: ML algorithms often require large datasets for satisfactory performance, but clinical data for cervical cancer diagnosis is often limited in size and quality. To compensate these limitations, studies applied various data pre-processing methods such as data augmentation [5, 7, 9, 10, 13, 14, 17, 18], image enhancement [4, 9, 27, 30], and the invention of image generation tools [33] to address imbalanced class distribution and small datasets. However, a general strategy is still required to address this issue.
(ii) Class Imbalance Problem: Imbalanced class distribution is a common issue in medical datasets, with classification models often favoring the majority class. Existing approaches are effective for binary classes but face limitations in multi-class classification tasks [7, 19, 29, 30].
(iii) Reliant on Pre-processing Interventions: Feature extraction is a crucial stage in conventional classification methods, with conventional feature extraction methods and pre-trained CNNs being commonly used in the literature. Hybrid feature learning approaches that combine deep learning and other machine learning algorithms have also been explored. However, limited research has been conducted on feature extraction for overlapping cells in raw medical images that may contain debris [4, 7, 14, 27, 29, 30]. Furthermore, while recent studies have achieved excellent detection and classification results, there is still a requirement for a computationally efficient cell segmentation method to be placed in the pipeline to accurately locate the region of interest and improve cancer detection accuracy [4, 9, 13, 17].
(iv) Generalizability of Models: The majority of the research has centered around classification and detection models evaluated on a single dataset [4, 5, 9–11, 13, 17, 18, 20, 27, 28, 30, 31]. The models must be validated over a variety of datasets and benchmark models to increase their generalizability.

## Methodology

This study evaluated and compared 13 CNN models with transfer learning on the public Herlev dataset for seven-class classification of cervical cancer cells. This section presents the dataset and methodology implemented in this study.

### Datasets

This study used the Herlev [21] database, which is openly accessible and contains 917 pap smear images that are unevenly distributed over seven different classes of cervical cells. The Herlev dataset can be retrieved from: https://mde-lab.aegean.gr/downloads.

**Fig. 1:**
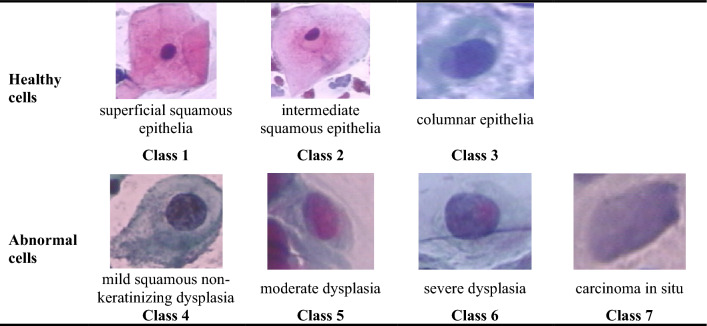
Samples of Herlev dataset in seven categories

**Fig. 2:**
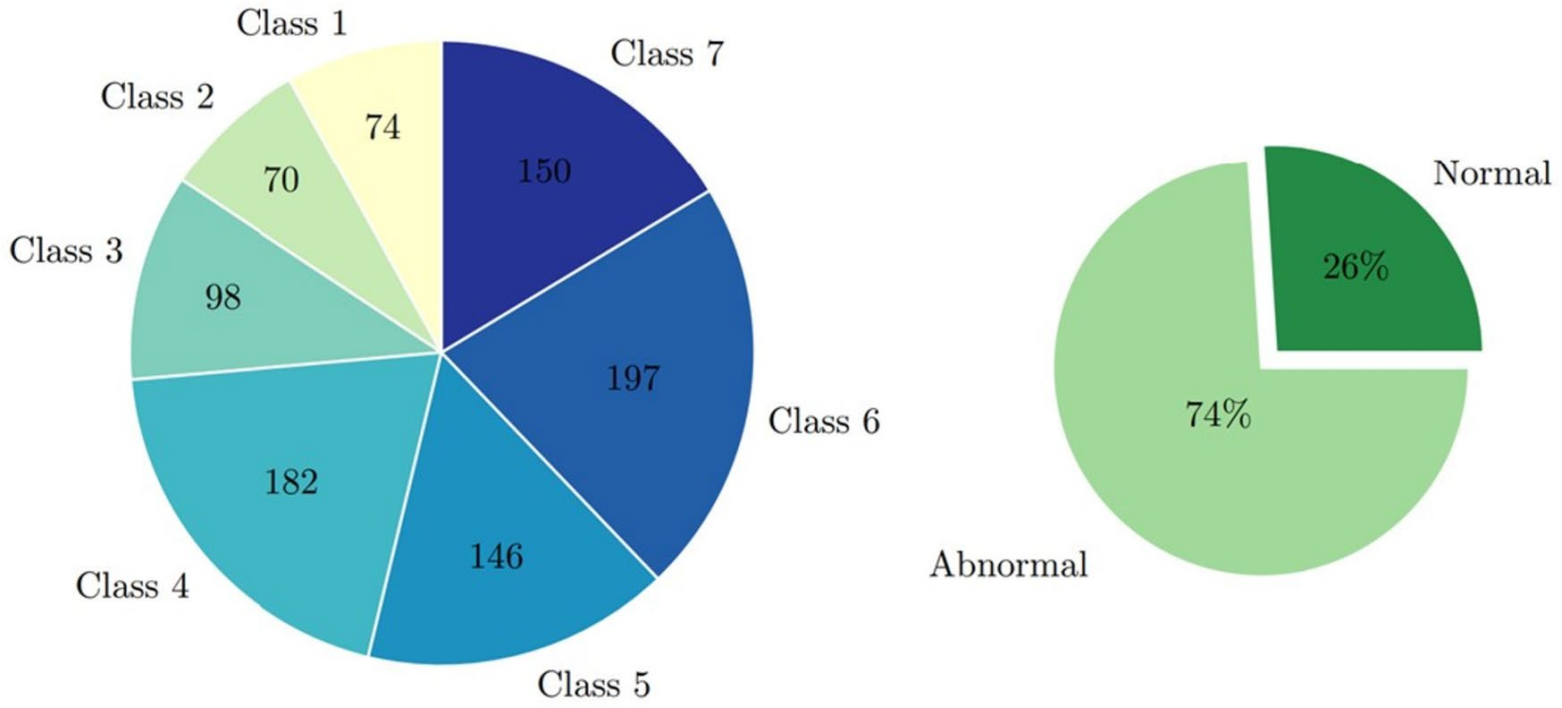
Distribution of the Herlev dataset

Figure 1 gives an example of the Herlev dataset in seven classes. Among these seven classes, the superficial squamous epithelia, intermediate squamous epithelia, and columnar epithelia belong to normal cells, whereas the others correspond to malignant cells. The cell types are sorted from normal to abnormal cell levels, with carcinoma in situ being the highest-grade lesion in the Herlev dataset. Figure 2 gives the distribution of the Herlev dataset.

### Data Pre-processing

As input to the CNNs, the Herlev database is loaded and transformed into an array that describes the shape of the input data. On top of that, a high degree of data variability is not ideal for model convergence. We normalized the inputs by scaling them into values between 0 and 1 to help the models generalize more rapidly and produce better results. To prevent over-fitting problem, the data are partitioned into a training set (80%), validation set (20%), and testing set (10%). Testing data are used to validate the models after they have been validated using training data.

### Methods

**Fig. 3:**
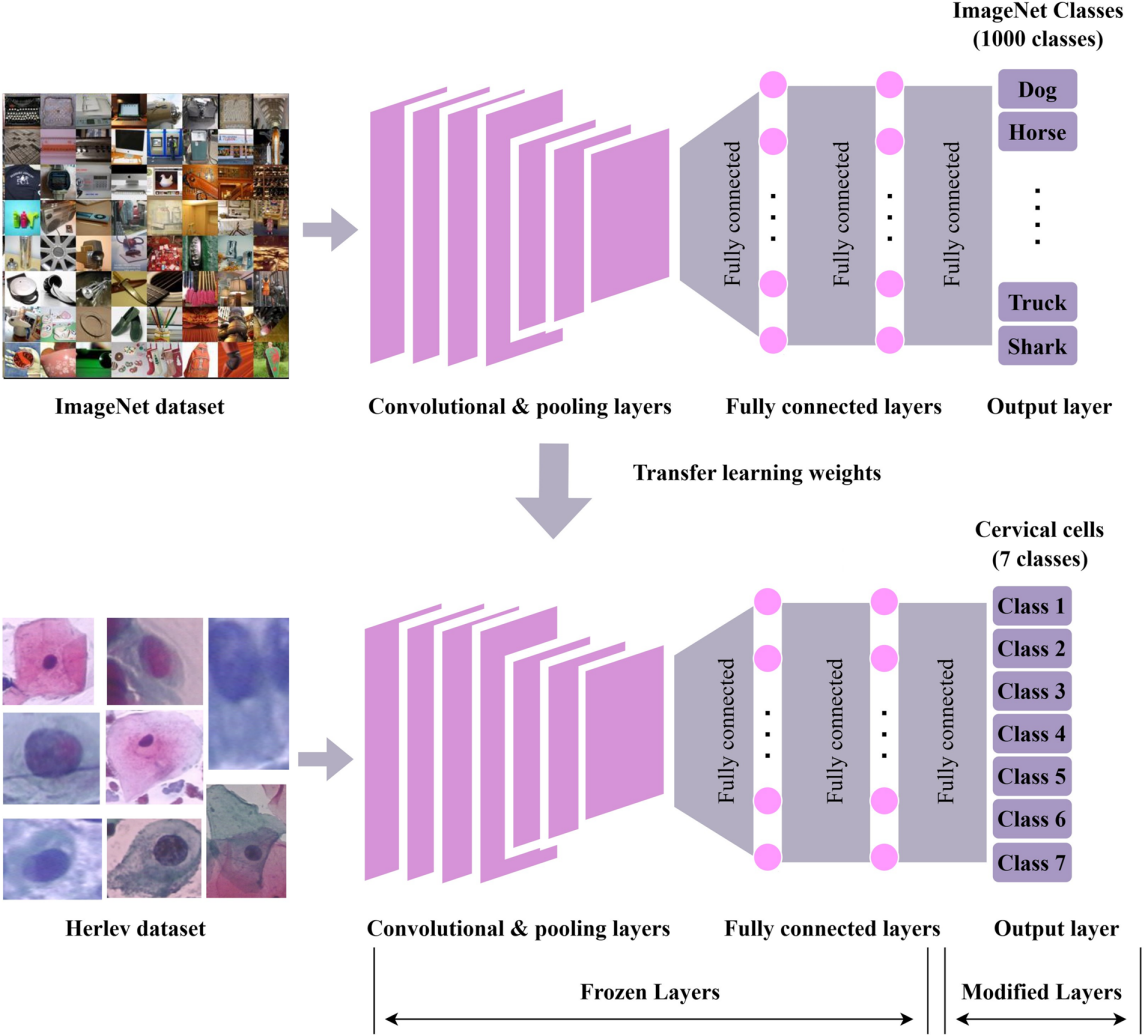
The workflow for cervical cancer classification using CNN with transfer learning

For classifying the cervical classes in this study, a transfer learning technique is employed. Transfer learning is referred to as the ability to apply knowledge and ability acquired from past work to new tasks. Customizing new CNN models from scratch requires enormous amounts of data for training as it is required to learn from millions of weights. However, it is a popular approach to automatically extract features from a new dataset using a pre-trained model. Each pre-trained model’s fully connected layers were replaced by modified fully connected layers with seven output nodes representing the seven cervical classes. Figure 3 gives an overall workflow for cervical cancer classification in this study.

After extensive research and review of the existing literature in the study of cervical cancer, CNN models were found to be the most widely utilized supervised ML techniques. CNN is typically used to handle data with grid pattern, like images. In [25], the authors concluded that CNN does not demand meticulous extraction of fine features and manual segmenting tumors or organs, but CNN demands graphical processing units (GPUs) for the model training phase because it is more computationally expensive and requires large volumes of data.

It has now become simpler to train DL for image classification tasks thanks to the growing amount of image datasets and computing power. In the area of cervical cancer diagnosis, the authors in [3–5], and [8] showed how deep learning may be applied and its implication for analyzing cervical images that are complex in nature and to tackle observer biases. These recent works of literature had motivated us to investigate the potent potential of the widely used CNN architectures for image processing—VGG-16, VGG-19, DenseNet-121, DenseNet-169, DenseNet-201, ResNet-50, ResNet-101, ResNet-152, Inception, Xception, MobileNet, and MobileNet-v2. These models were chosen for their robust performance in a range of classification tasks.

**Fig. 4:**
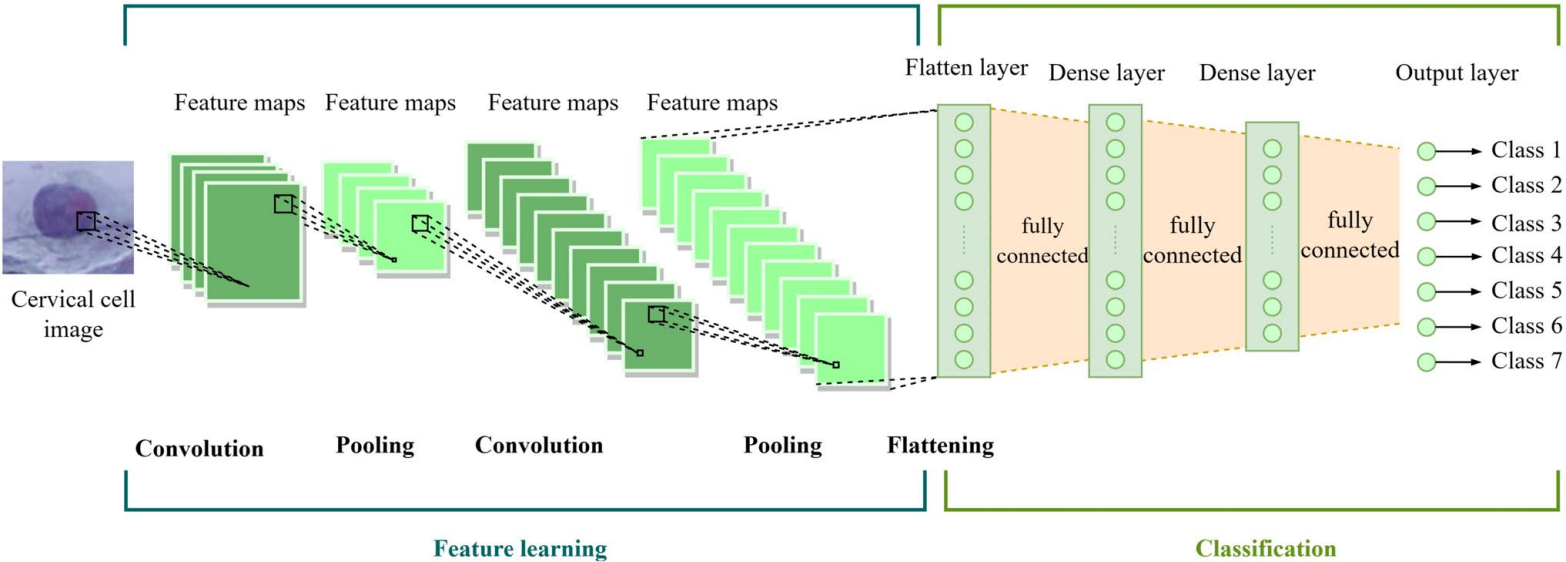
The shared fundamental network structure of the CNN models employed in this study

These subsequent models are either built upon or enhanced versions of the initial vanilla CNN. Figure 4 illustrates the common fundamental components shared by these CNN models. These components are represented mathematically and discussed in Table 1.

**Table 1:** Common core components of a CNN model

Core component	Mathematical expression	Details
Convolutional layer	${\mathrm{Conv}}_{\mathrm{i}}={\upsigma }_{\mathrm{i}}\left({W}_{i}*{X}_{i}+ {b}_{i}\right)$ where ${X}_{i}$ represent the input to the $i$-th convolutional layer, ${W}_{i}$ represent a set of filters for the $i$-th convolutional layer, ${b}_{i}$ represent the bias term for each filter, $*$Represent the convolution operation, and ${\mathrm{Conv}}_{\mathrm{i}}$ represent the output feature maps from the $i$-th convolutional layer	Function: Extract features Input: 3D tensor (height, width, and channels of the data) Output: Feature maps
Pooling layer	${\mathrm{Pool}}_{i}\left[j, k, l\right]=\mathrm{max}\left({\mathrm{Conv}}_{i}\left[{S}_{ij}: \left({S}_{ij}+{K}_{i}\right), {S}_{ik}: \left({S}_{ik}+{K}_{i}\right), l\right]\right),$ where ${\mathrm{Conv}}_{i}$ represent the input feature maps to the $i$-th pooling layer, $\mathrm{max}\left(\right)$ represent the pooling operation, ${K}_{i}$ represent the pooling size for the $i$-th pooling layer, ${S}_{i}$ represent the stride for pooling, and ${\mathrm{Pool}}_{i}$ represent the output feature maps after pooling from the $i$-th pooling layer	Function: Downsample the feature maps Input: Feature maps from the $i$-th convolutional layer Output: Downsized feature maps
Fully connected layer	${\mathrm{FC}}_{i}={\sigma }_{{FC}_{i}}\left({W}_{{FC}_{i}}*{F}_{i}+{b}_{{FC}_{i}}\right),$ where ${F}_{i}$ represent the flattened input vector to the $i$-th fully connected layer, ${W}_{{FC}_{i}}$ represent the weight connecting the previous layer to the $i$-th fully connected layer, ${b}_{{FC}_{i}}$ represent the bias term for the $i$-th fully connected layer, ${\sigma }_{{FC}_{i}}$ represent the activation function, and ${\mathrm{FC}}_{i}$ represent the output of the $i$-th fully connected layer	Function: Establishing connections between each neuron in the previous layer and the neurons in the current layer Input: Flattened vector Output: A vector representing the final prediction of the model

VGG based on AlexNet was proposed to address the depth of CNNs. VGG employs $1\times 1$ convolutional layers to increase the decision function's non-linearity without compromising the receptive fields. VGG can have a lot of weight layers because of the 3 × 3 small convolution filters and having more layers will result in better performance. In the VGG network architecture, the number of filters along with each stack of the convolutional layers make it a large network and this requires more time to train its parameters. This study experimented with the VGG-16 and VGG-19 models that comprise of 16 and 19 convolutional layers, respectively

DenseNet is a variant of CNN composed of Dense Blocks forming dense connections to directly connect all layers. To ensure a feed-forward configuration and transfer collective knowledge to subsequent layers, each layer within the network receives inputs from all earlier layers while simultaneously imparting its own feature maps to those layers. Thus, this architecture offers improved computational efficiency and memory efficiency. This study experimented with DenseNet-121, DenseNet-169, and DenseNet-201 which comprises of four dense blocks. DenseNet-121 has (6, 12, 24, 16) layers, DenseNet-169 has (6, 12, 32, 32) layers whereas DenseNet-201 has (6, 12, 48, 32) layers in the four dense blocks.

ResNet is a variant of CNN that is made up of residual blocks. The core of residual blocks is the skip or shortcut connection which can overcome the vanishing gradient drawbacks by allowing this alternate path for the information to flow from one layer to the next layer after the immediate next [6]. This study experimented with ResNet-101, ResNet-152, and ResNet-50 with 101, 152, and 50 layers, respectively.

Inception is a CNN design that was created to address the issue induced by complicated and deep networks. The Inception architecture employs parallel layers, leading to a broader network architecture instead of a deeper one. Xception is a CNN architecture that is based on Inception and relies on modified depthwise separable convolution layers in which a $1\times 1$ convolution is performed prior to any $n\times n$ spatial convolutions.

MobileNet uses depthwise separable convolutions and it is designed to be used in mobile applications. Compared to networks using conventional convolutions of the same depth, MobileNet has significantly fewer parameters and lower latency. It is recognized as one of the most compact CNN architectures. This study experimented with MobileNet and MobileNet-v2. MobileNet-v2 is an improved version of the MobileNet that integrates linear bottlenecks between the layers and has introduced a shortcut path to the bottlenecks to speed up training and improve accuracy.

Table 2 provides a concise overview of the essential characteristics and fundamental operations of each model. In the table, ${H}_{i}$ represents the feature maps generated at layer $i$, ${X}_{i}$ denotes the input to layer $i$, $\sigma$ refers to the activation function, and ${W}_{i}$ and ${b}_{i}$ represent the weights and biases, respectively.

**Table 2:** Overview and fundamental operations of the selected CNN models

Model	Main features	Mathematical expression of the fundamental operations
VGG-16, VGG-19	Deep architecture Made up of convolutional layers with small filter size	Convolutional layer Pooling layer Fully connected layer
DenseNet-121, DenseNet-169, DenseNet-201	Introduced dense blocks that allow feed-forward connectivity to every other layer Allows feature reuse as the feature map size remains unchanged inside the dense block A solution to vanishing gradient problem as the gradients pass directly through dense connections	Dense block: ${H}_{i}={\sigma }_{i}\left(\left[{H}_{i-1}, ..., {H}_{0}\right]\right)$ Transition layer: ${H}_{i}=\sigma \left({W}_{i}*{H}_{i-1}+{b}_{i}\right)$
ResNet-50, ResNet-101, ResNet-152	Introduced residual learning based on skip connections Deep architecture Minimize model dimension and maximization of representational power via bottleneck	Residual block: ${H}_{i}={H}_{i-1}+\sigma \left({W}_{i}*\sigma \left({W}_{i-1}*{H}_{i-1}+{b}_{i-1}\right)+{b}_{i}\right)$
Inception	Introduced inception block which allows multiple filter sizes and pooling operations in parallel Operates in lower depths	Inception module: ${H}_{i}=\left[1\times 1 \mathrm{Conv},3\times 3 \mathrm{Conv},5\times 5 \mathrm{Conv},\mathrm{Pool}\right]*{X}_{i},$ where $1\times 1 \mathrm{Conv},3\times 3 \mathrm{Conv},$ and $5\times 5 \mathrm{Conv}$ refers to a $1\times 1$, $3\times 3$ and $5\times 5$ convolution operation applied to the input feature maps, respectively
Xception	Reduced computational complexity through depthwise convolution followed by a pointwise convolution Using blocks of depthwise separable convolution in parallel	Depthwise separable convolution: ${H}_{i}=\sigma \left({DW}_{i}*\left({PW}_{i}*{X}_{i}\right)\right),$ where $D$ and $P$ denotes depthwise convolution and pointwise operations, respectively
MobileNet, MobileNet-v2	Shallow network architectures Use of depthwise separable convolution Suitable for low-powered devices	Depthwise separable convolution: ${H}_{i}=\sigma \left({DW}_{i}*\left({PW}_{i}*{X}_{i}\right)\right),$ where $D$ and $P$ denotes depthwise convolution and pointwise operations, respectively

**Fig. 5:**
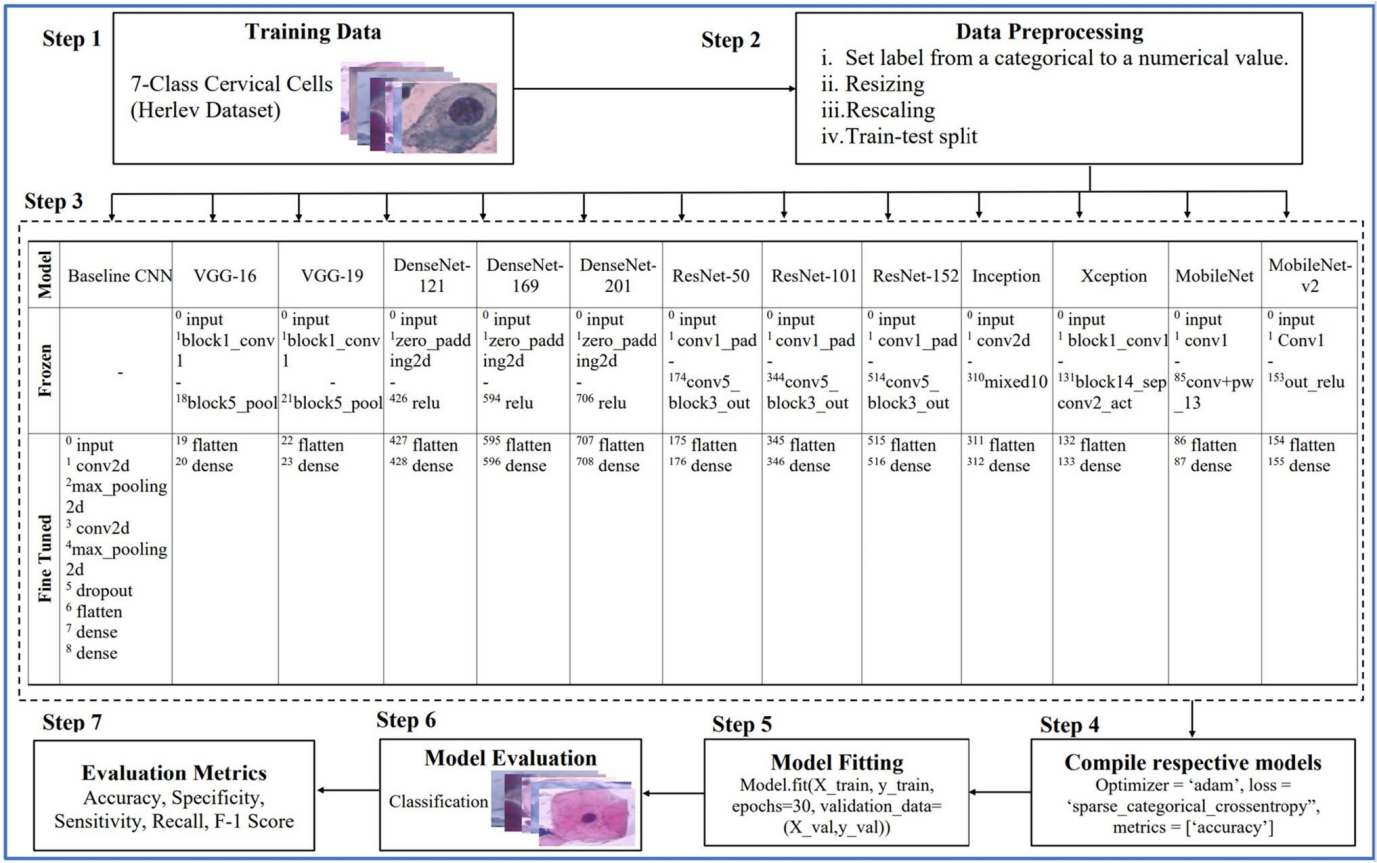
Description of the experimental working flow

Figure 5 gives a detailed description of the flow of the experiment. In step 3 of the figure, the superscripts indicate the specific layer within the model architecture. For instance, the CNN model's initial layer is indicated by “0” while the fifth layer is denoted by “5”. The pseudocode of the CNN models with transfer learning is presented in Algorithm 1. The model takes the public Herlev dataset as input and produces class labels as output. Subsequently, the predicted class labels are evaluated against the ground truth (actual labels), and metrics including accuracy, specificity, sensitivity, recall, and F1-score are determined.

**Algorithm 1:**
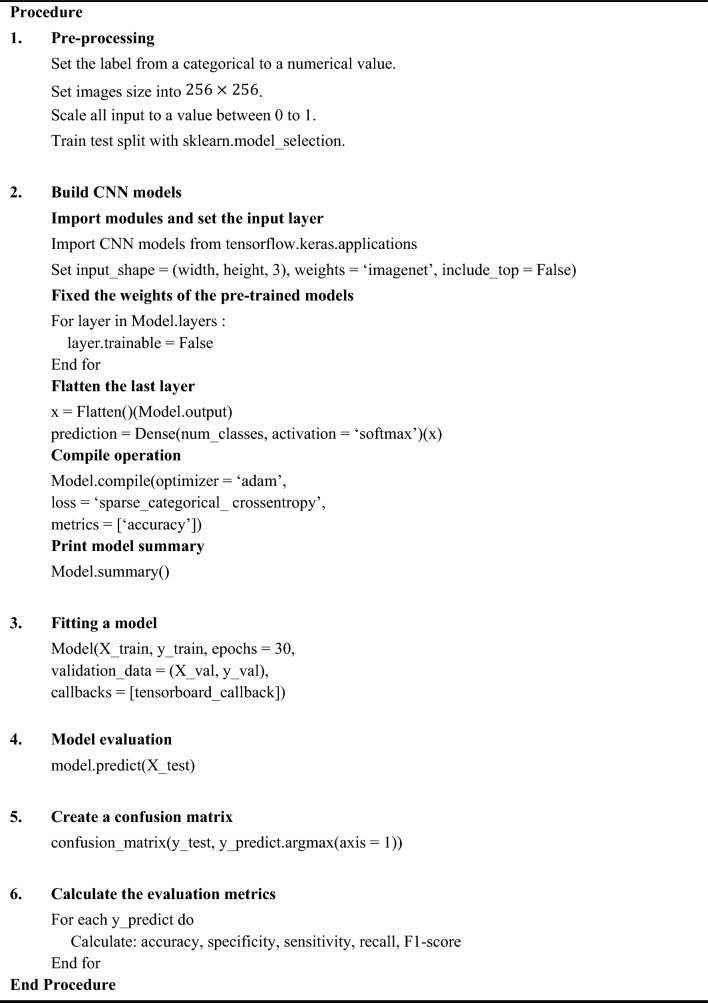
Pseudocode for cervical cancer cells classification using CNN models with transfer learning.

### Experimental Settings

VGG-16, VGG-19, DenseNet-121, DenseNet-169, DenseNet-201, ResNet-50, ResNet-101, ResNet-152, Inception, Xception, MobileNet, and MobileNet-v2 were employed as training models for the Pap images from the publicly available Herlev dataset. These models were selected because of their exceptional performance on several detection and classification tasks demonstrated in the surveyed literature. The seven cervical classes were therefore classified using a transfer learning method.

Table 3 outlines the hyperparameters used to train the CNN models and these were determined after a thorough review of the literature. The last layers were altered to a customized fully connected layer made up of seven neurons compatible with the seven classes because this is a seven-class classification task. At the output layer, Softmax activation functions are specified. This function normalizes the outputs, transforming them from weighted sum values into the probability of membership for each class. The Softmax activation function can be represented by Eq. (1).

1 $${p}_{i}=\frac{\mathrm{exp}\left({x}_{i}\right)}{{\sum }_{j=1}^{K}\mathrm{exp}\left({x}_{j}\right)},$$

where $\mathrm{exp}\left({x}_{i}\right)$ represents the exponential function applied to the input vector, $\mathrm{exp}\left({x}_{j}\right)$ represents the exponential function applied to the output vector, and $K$ refers to the number of classes.

**Table 3:** Hyperparameters settings used for the pre-trained models

Hyperparameter	Parameter setting
Input size	$256\times 256\times 3$
Batch size	$32$
Epoch	$30$
Activation function	Softmax
Optimizer	Adam
Learning rate	$0.001$
Loss function	Sparse categorical cross-entropy

A loss function, an optimizer, and selected evaluation measures are then used to evaluate the models. Loss is computed using sparse categorical cross-entropy. Improved training and test results are indicated by a decreased loss function value. Equation (2) shows the loss function employed in this study:

2 $${\text{Loss}} = - \mathop \sum \limits_{i = 1}^{K} q_{i} \log p_{i} ,$$

where ${q}_{i}$ denotes the true label, and ${p}_{i}$ denotes the probability associated with the $i$-th class. Then, Adam optimizer is applied to optimize the input weights by comparing the prediction.

The weights of the remaining layers have already been fine-tuned since the CNN models were previously trained using the publicly accessible ImageNet dataset, leaving just the customized output layers that require training. Accordingly, a batch size of 32 and a total of 30 epochs were set for the training to ensure convergence. Additionally, the selection of the number of epochs is also in reference to [6] and [10].

### Experimental Environments

The Keras library based on TensorFlow 2.8.2, an open-source Python deep learning library, has been used to implement the CNN architectures. The training of the network was implemented in Google Collaboratory which supports free access to NVIDIA Tesla T4 GPU, a graphic driver with version 460.32.03, and a CUDA 11.2 version.

### Evaluation Metrics

The output of the publicly available deep CNN models namely, VGG-16, VGG-19, DenseNet-121, DenseNet-169, DenseNet-201, ResNet-50, ResNet-101, ResNet-152, Xception, MobileNet, and MobileNet-v2 were examined. An unknown set of testing data is provided to a classifier after training it with training data to check if it can accurately classify the samples. This study used accuracy, specificity, recall, sensitivity, and F1-score as the evaluation measures as these are among the most frequently used evaluation metrics to examine multi-class classification efficiency. The mathematical expression for the selected metrics is outlined in Table 4.

**Table 4:** Mathematical expression of the selected metrics

Metrics	Formula
Precision	$\frac{TP}{TP+FP}$
Recall/Sensitivity	$\frac{TP}{TP+FN}$
F1-score	$\frac{2\times \mathrm{Precision}\times \mathrm{Recall}}{\mathrm{Precision}+\mathrm{Recall}}$
Specificity	$\frac{TN}{TN+FP}$
Accuracy	$\frac{TP+TN}{TP+TN+FP+FN}$

*TP* true positive, *TN* true negative, *FP* false positive, *FN* false negative

## Experimental Results

### Results of the Pre-trained Classifier Models

In this work, the results of a baseline CNN model versus that of twelve other CNN models employing transfer learning were assessed and compared. Table 5 displays the performance comparison of all the models. To provide a clear reference to the top-performing CNN models, the best results were visually distinguished by bold formatting.

**Table 5:** Performance metrics for the respective models

Model	Training duration (mins)	Accuracy	Precision	Recall	Specificity	F1-score	Sensitivity
Baseline CNN	**00:48.3**	0.8382	0.5415	0.5244	0.9021	0.5054	0.5244
VGG-16	03:16.9	0.8229	0.5016	0.4634	0.9063	0.4205	0.4634
VGG-19	04:23.4	0.8382	0.5415	0.5244	0.9021	0.5054	0.5244
DenseNet-121	01:58.5	0.8473	0.5346	0.5488	0.9051	0.5374	0.5488
DenseNet-169	02:30.1	0.8526	0.5755	0.5854	0.9077	0.5783	0.5854
DenseNet-201	02:49.6	**0.8702**	0.6226	**0.6341**	0.9151	0.6165	**0.6341**
ResNet-101	03:31.5	0.7445	0.0396	0.1341	0.8808	0.0434	0.1341
ResNet-152	05:30.2	0.7496	0.1134	0.1585	0.8839	0.0801	0.1585
ResNet-50	02:26.2	0.7145	0.1463	0.2317	0.7839	0.1015	0.2317
Inception	02:26.2	0.8486	0.5709	0.5732	0.9049	0.5705	0.5732
Xception	02:38.2	0.8672	**0.6320**	0.6220	**0.9168**	**0.6214**	0.6220
MobileNet	01:24.7	0.8611	0.6072	0.5976	0.9133	0.5961	0.5976
MobileNet-v2	00:57.5	0.8345	0.5061	0.5122	0.9036	0.5020	0.5122

### Results of the Multi-class Classification Task

**Fig. 6:**
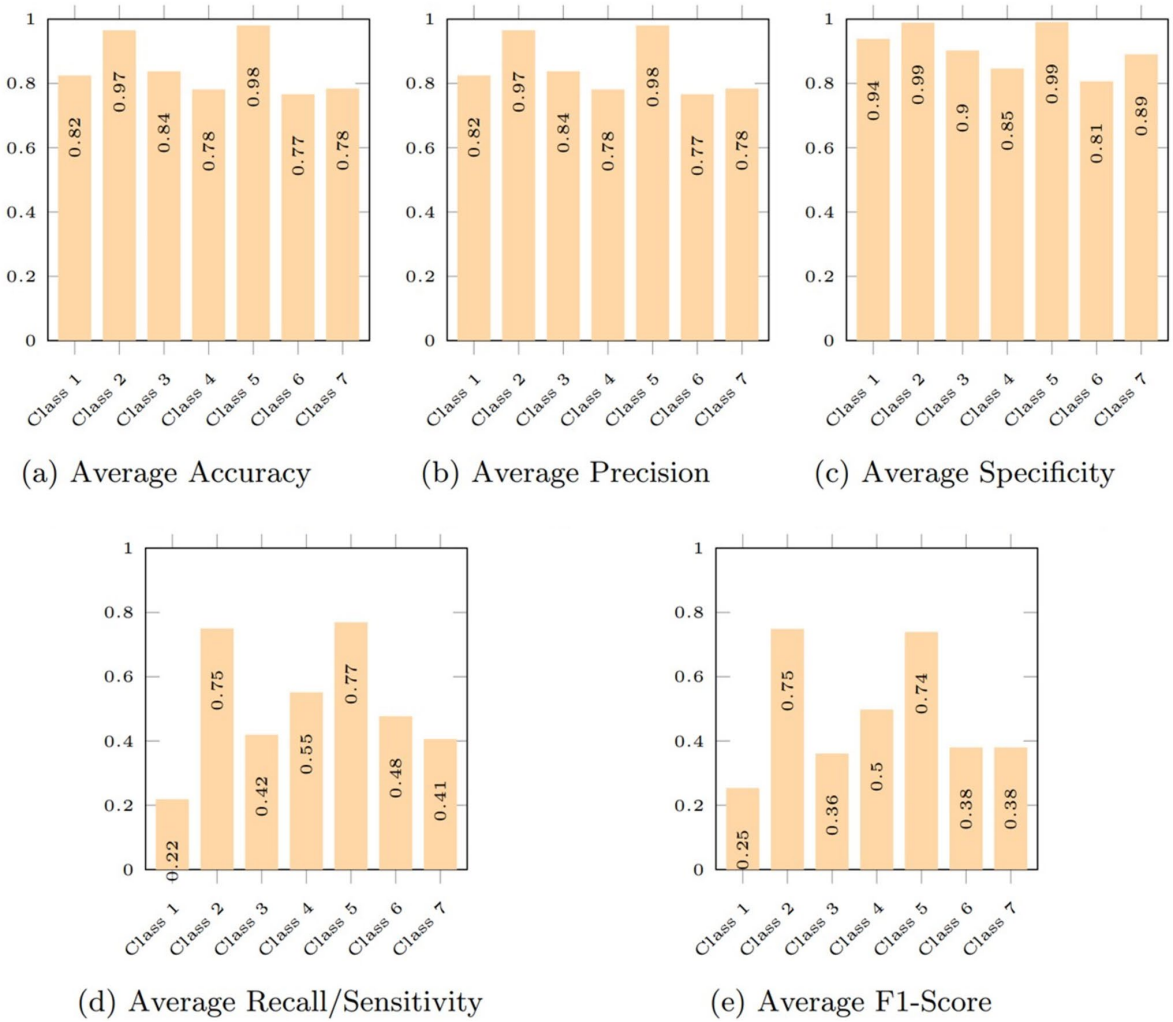
Comparison of average metrics obtained for each class

**Fig. 7:**
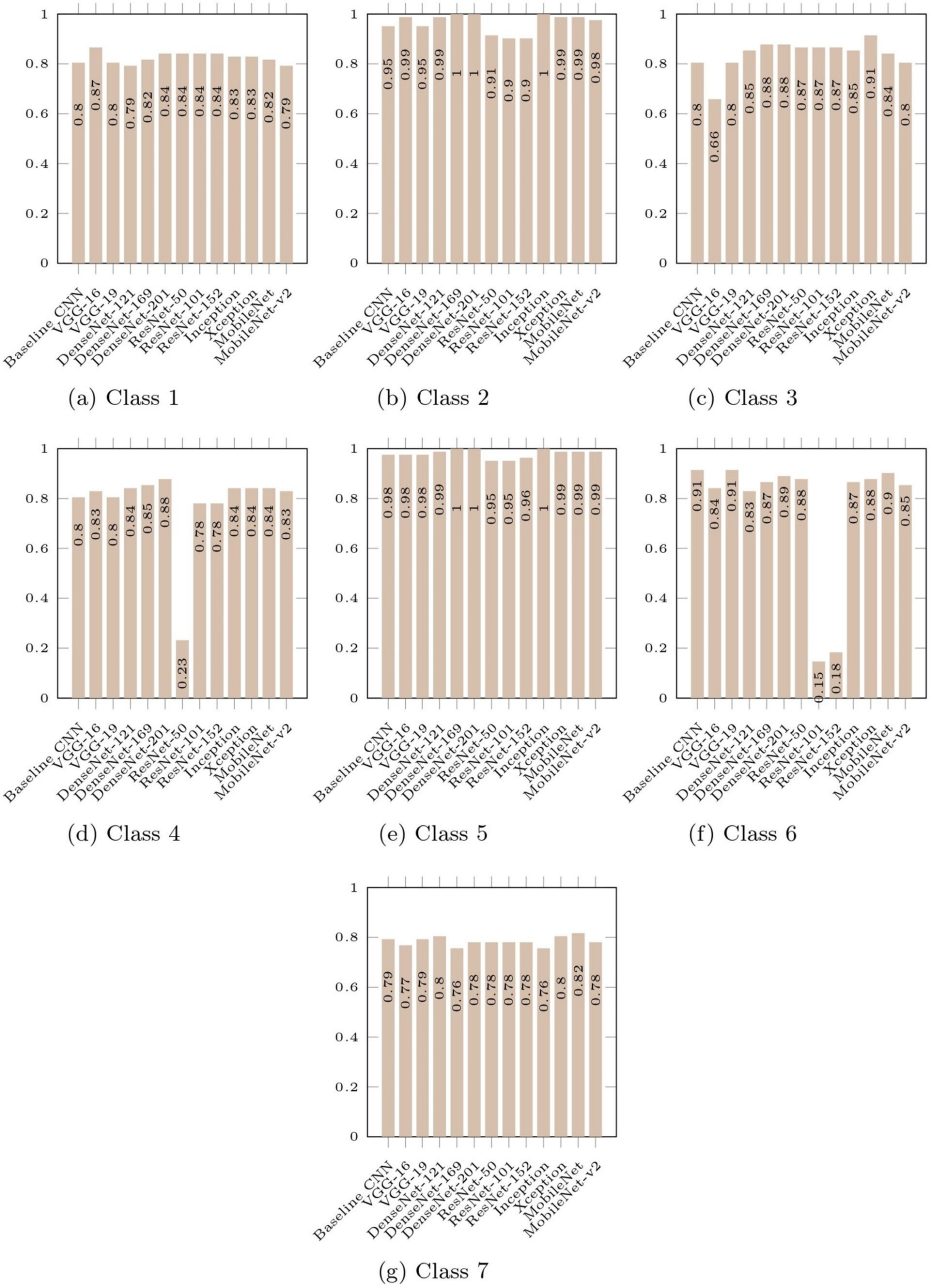
Comparison of class accuracy obtained for each model

The metrics for each model are averaged with respect to the cell classes in Fig. 6 to assess the overall robustness of pre-trained CNN models for multi-class classification. This will help us understand the correlation between cell type, class distribution, and classification performance. To evaluate each model’s functionality, the accuracy of the models relative to the classes in Fig. 7 is provided.

## Analysis and Discussion

### Evaluation of the Pre-trained CNN Models

The evaluation metrics and the computation time for the 13 CNN models used in this study were analyzed and examined in this section. Next, the imbalance class problem on classification tasks is validated. Lastly, a comparison study is conducted to study the differences in the performance of all the models that were studied.

#### Performance of the Pre-trained CNN Models

It should be noted that this study, like others in the same field, faces a common challenge, which is the lack of specified performance thresholds for detecting cervical cancer. The existing literature does not yet provide a target accuracy for classifying images related to cervical cancer screening. In [34], the authors concluded that guidelines from screening programs and professional organizations lack cited evidence supporting the performance metrics for cervical cancer tests. For example, the United Kingdom’s Office for Health Improvement & Disparities [35] set a performance threshold of a sensitivity greater than 90% for all abnormalities and a sensitivity greater than 95% for high-grade abnormalities, based on the accuracy of the initial cytology examination as determined by rapid review. On the other hand, the Canadian Partnership Against Cancer [36] set a performance threshold of at least 65% for percentage of positive Pap tests that are confirmed to have pre-cancerous lesions or invasive cancer within a span of 12 months. However, these thresholds are inconsistent and lack cited evidence. Nevertheless, according to Foody [37], a widely accepted target for image classification accuracy is considered to be at least 85%. Hence, the results obtained in this study, which surpass 85%, can be considered broadly acceptable. However, it is important to emphasize that accurate cancer detection is crucial and a matter of life and death, and therefore further improvement is indeed necessary. However, this current work does not delve into enhancing or improving the performance of the models as that is beyond the scope of this study.

Pre-trained networks were used in this study to get around the need for a massive number of datasets for training of CNN models. Results presented in Table 5 show that DenseNet-201 attained a superior accuracy of 87.02% and outperformed the ResNet-50, ResNet-101, ResNet-152, VGG-16, MobileNet-v2, Baseline CNN, VGG-19, DenseNet-121, Inception, DenseNet-169, MobileNet, and Xception models in terms of accuracy. All models except ResNet-50, ResNet-101, and ResNet-152 achieved accuracies of over 80%. Out of the models experimented, only four achieved accuracy levels above 85%. These models are DenseNet-169 with an accuracy of 85.26%, MobileNet with an accuracy of 86.11%, Xception with an accuracy of 86.72%, and DenseNet-201 with the highest accuracy of 87.02%.

**Fig. 8:**
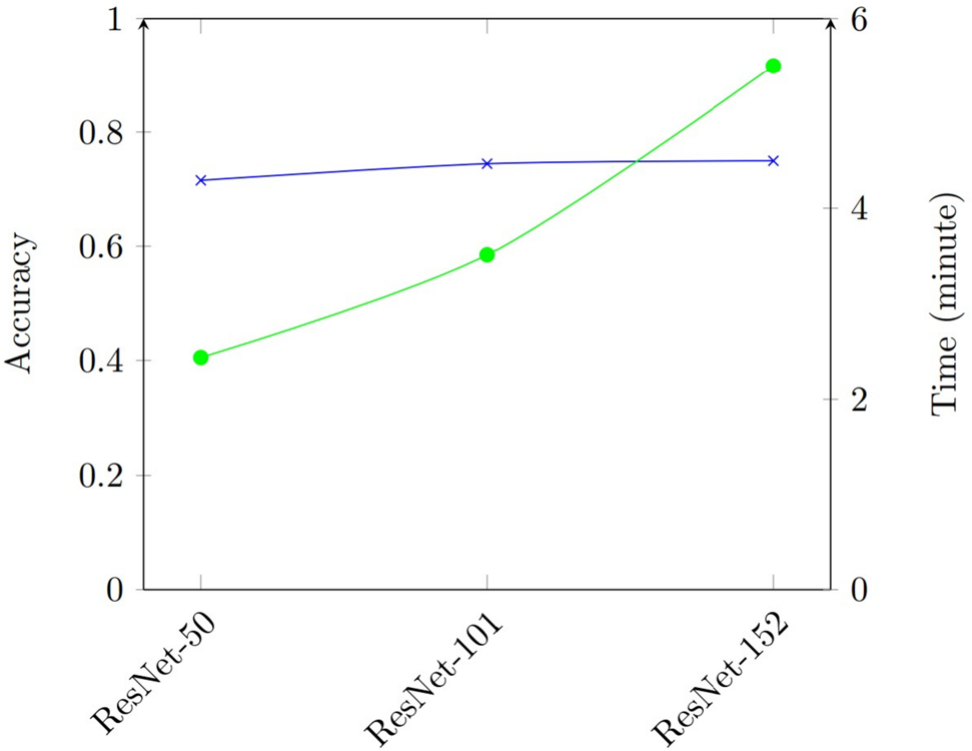
Comparison of accuracy and training time of the ResNet models

On the other hand, it was found that the three ResNet models in this study had accuracy values in the range of 71.45% and 74.96%, which were lower than the other models. The experimental results depicted in Fig. 8 show that ResNet-152 performs just slightly better than ResNet-50 and ResNet-101, despite taking a significantly longer training period. This implies that some of the layers may be unnecessary. This result is in line with the findings in [33] that one of the drawbacks of ResNet is that it preserves information from layers but many of these layers may provide little to no information.

As mentioned earlier, the highest accuracy was obtained by DenseNet-201. The network merges the features of the prior levels rather than adding them. To that end, DenseNet can eliminate the difficulties with vanishing gradient, improve feature propagation, allow feature reuse, and require significantly fewer parameters. Additionally, the top three performing models of MobileNet, Xception, and DenseNet-201 have exhibited great specificity and sensitivity. These models outperformed the ResNet models while having a less complex network architecture. This implies that a deep-layer network would not always be the best approach and might potentially cause the performance to deteriorate.

#### Time Complexity Comparison

**Fig. 9:**
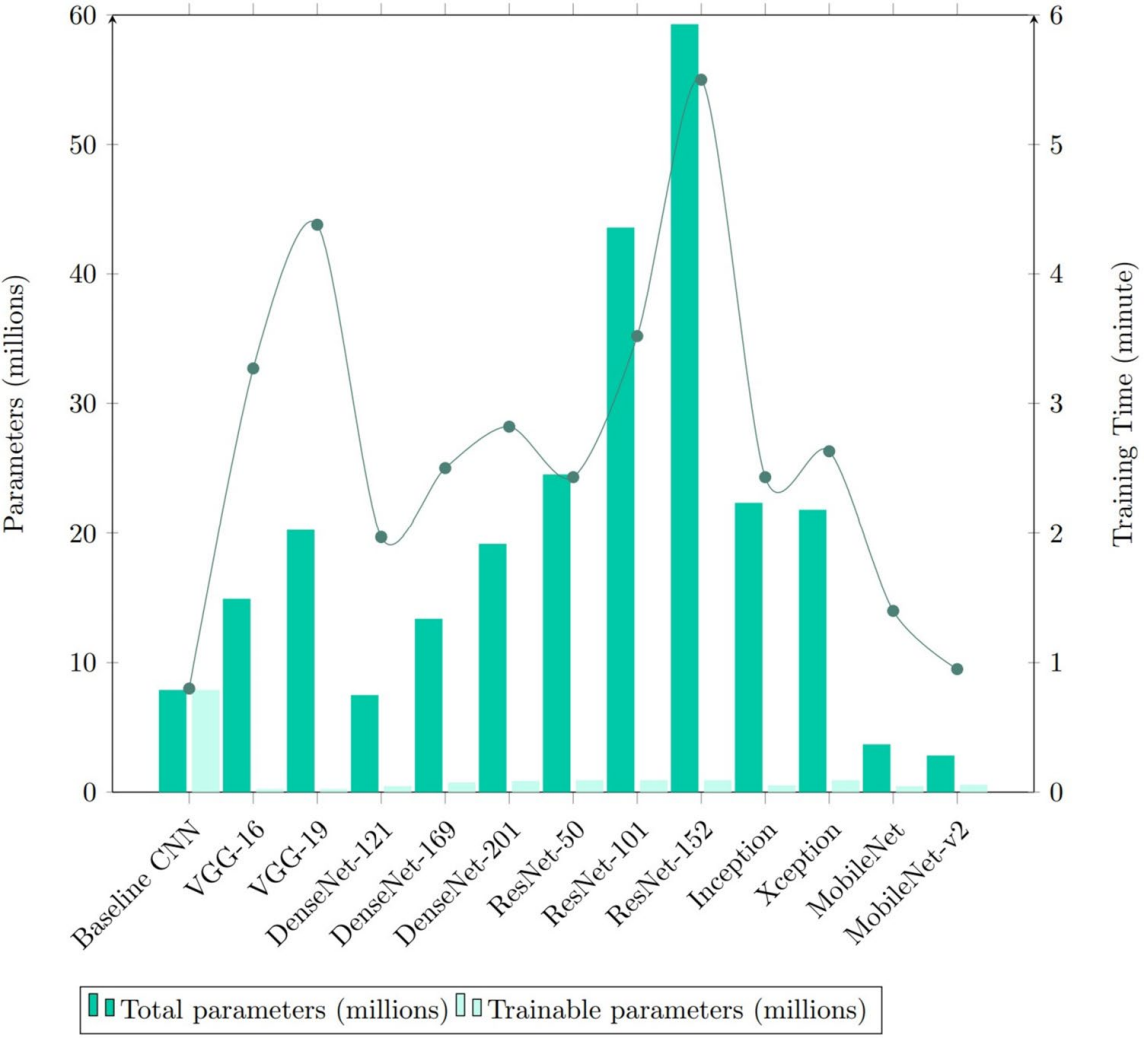
Comparison of number of total parameters, number of trainable parameters and training time (minutes) taken for each model

The comparison of model sizes and how this varies with the time required for training is shown in Fig. 9. It was discovered that ResNet-152 which is the largest model and the one with the greatest number of parameters overall, required the greatest time to train (5.50 min). On the other hand, the baseline CNN was observed to have the shortest training time due to its small and shallow model architecture. Moreover, the training times for MobileNet and MobileNet-v2 were just 1.4 min and 0.95 min, respectively. The idea that inspired the development of the MobileNet and MobileNet-v2 models was to efficiently maximize accuracy on the constrained resources available for an embedded or on-device application. Therefore, MobileNet and MobileNet-v2 are both shallow deep neural networks that can train very quickly due to the absence of several training parameters. To our knowledge, this is the first examination on the suitability and performance of MobileNet and MobileNet-v2 for cervical cancer screening.

#### Limited Dataset and Imbalance Class Problems

Due to the limited dataset employed in this study, which contains only 917 images in total, the model accuracies were encouraging but not particularly impressive and can still be further improved. The distribution of the seven classes was found to be unevenly distributed, indicating that the data is unbalanced. Only 26.39% of the images were classified as normal cells. This is a prevalent problem in the domain of medical imaging analysis since biological datasets are typically imbalanced and there are usually far more negative samples than positive ones [38].

In this experiment, the precision, recall, and sensitivity metrics were used to assess the problem of imbalanced data. Precision is sensitive to class imbalance since it takes into account the number of negative samples that have been incorrectly classified as positive. Table 5 shows that the top three performing models have a precision of between 0.62 and 0.64. These figures suggest that although the accuracy is encouraging, the precision is not favorable because it is impacted by data imbalance. Nevertheless, the recall values of these three top-performing models were found to be between 0.59 and 0.64. The exclusion of the number of negative cases misclassified as positive caused such impact on recall.

It is important to highlight that the pre-trained models utilized in this study were originally trained on the ImageNet dataset, which is built on real-world natural images, and that the characteristics of natural images differ significantly from those of unprocessed Pap smear images. Therefore, the performance of the models may not have shown substantial improvement or outperformed a custom CNN model. The experimental results and performance comparison obtained from this study lay the groundwork for future investigations in this field.

#### Comparative Studies

This study employed the Adam optimizer for 30 epochs with reference to the parameter settings in both [6] and [10]. The reason for these parameter selections is to be in line with the current trend in the existing literature and to compare the results with the existing studies presented in Table 6. It is worth noting that these may not be the ideal parameters to utilize, and additional tuning will certainly be necessary to achieve sophisticated performance, but they are sufficient to be used as preliminary results to lay the foundation for advanced research in this area. However, DenseNet-201 was not evaluated in both [6] and [10], hence we were unable to compare the model performance for this model. Additionally, the performance of the VGG-16, VGG-19, Inception, Xception, and MobileNet models in this study was found to have surpassed the performance obtained in [10]. The authors in [10] used their own dataset rather than the Herlev dataset and different model settings, which may have contributed to the differences in the results and findings.

**Table 6:** Comparison of accuracy with the results reported in [6, 10], and [33]

Models	Accuracy [this study]	Accuracy [6]	Accuracy [10]	Accuracy [33]
Baseline CNN	0.8382	–	–	–
VGG-16	0.8229	0.8337	0.6670	–
VGG-19	0.8382	0.8455	–	–
DenseNet-121	0.8473	–	–	–
DenseNet-169	0.8526	–	–	–
DenseNet-201	0.8702	–	–	0.9687
ResNet-101	0.7445	0.9045	–	–
ResNet-152	0.7496	–	–	–
ResNet-50	0.7145	0.8937	–	0.9554
Inception	0.8486	–	0.701	–
Xception	0.8672	–	0.731	–
MobileNet	0.8611	–	0.691	–
MobileNet-v2	0.8345	–	–	–

In [33], the authors also evaluated the performance of DenseNet-201 and ResNet-50 along with its proposed model with 100 epochs on the public Herlev dataset. These promising results could be primarily due to the contribution of the CCG-taming transformers-based cervical image-generating tool, which addressed the issue of uneven distribution of classes and data limitations. This finding proved that larger datasets with balance class distribution can significantly improve the results of the pre-trained models.

Overall, the pre-trained models are highly convincing and inspiring, thereby demonstrating the practicality of our proposal for automated cervical cancer diagnosis without relying on segmentation methods or hand-crafted features.

### Evaluation of the Multi-class Classification Task

This section focuses on evaluating the results of classifiers in multi-class classification. The performance metrics for each class are analyzed to identify any patterns of strong or weak performance across different classes. Subsequently, the performance of each model is assessed in relation to individual classes.

#### Class-by-Class Performance Comparison

As shown in Fig. 6, Class 5 obtained the highest average accuracy, average recall, average specificity, and average sensitivity of all the classes, except for the average F1-score and average precision. This suggests that every pre-trained model evaluated in this study is competent at correctly classifying Class 5. Next to Class 5, the average accuracy, recall, specificity, and sensitivity of Class 2 were second-best overall. In addition, the average F1-score and average precision for Class 2 were shown to be greater than those of Class 5 as seen in Fig. 6.

**Fig. 10:**
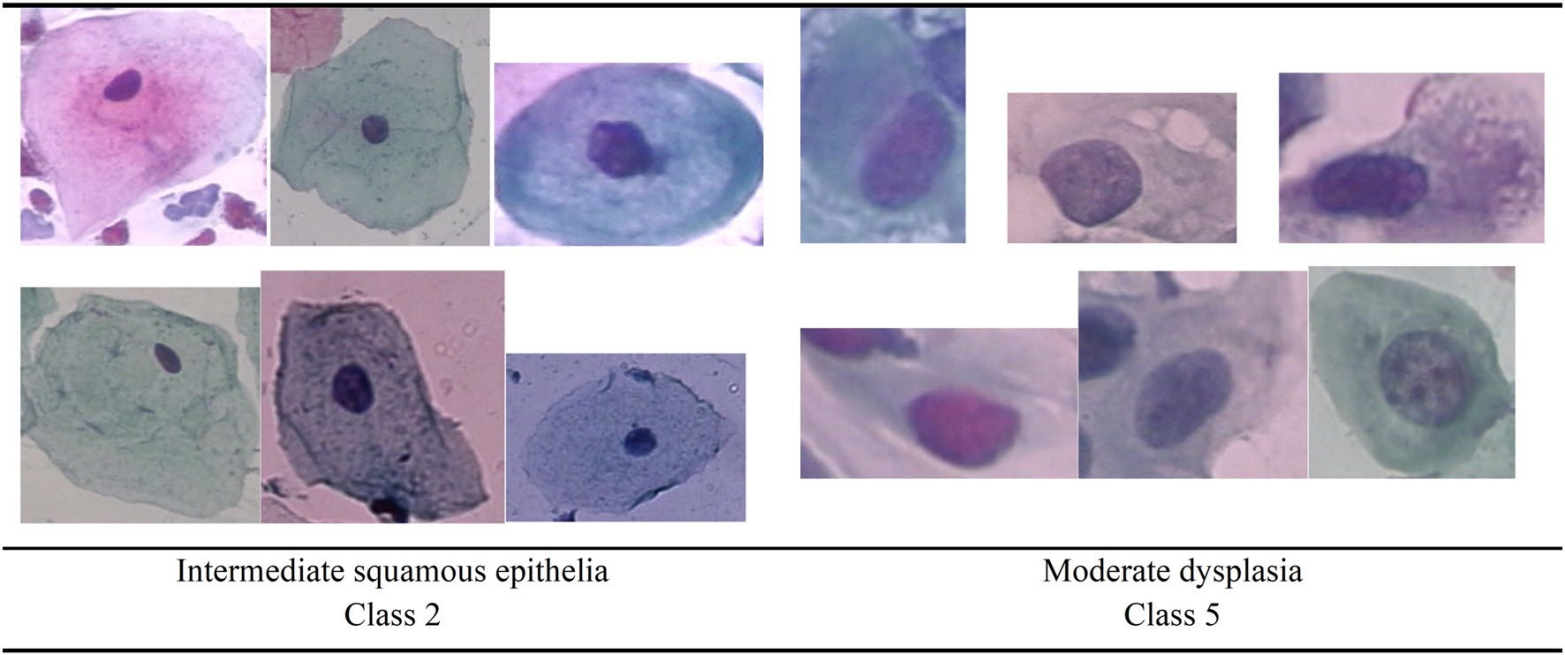
Samples of Class 2 and Class 5

To explore the physical and visual characteristics of Class 5 and Class 2, random samples were chosen from each class and presented in Fig. 10. We discovered that the attributes of these two classes are remarkably similar. They both were round in shape and have a large and dark nuclei. This may be the cause of the relative success of our models for these two classes in particular. These classes were probably easier to recognize than the other classes because of their visual attributes.

Moreover, it is worth noting that Class 5 is superior to the other classes in terms of sensitivity (0.7692) and specificity (0.9091). The findings imply that the pre-trained models showed significant performance in accurately classifying Class 5 while simultaneously excluding the negative samples. Additionally, Class 2 revealed similar findings.

Contrastingly, it was discovered that all the abnormal cell classes, except for Class 5, obtained unsatisfactory classification metrics values with accuracy lower than 0.80 and are significantly inferior to the normal classes. This suggests that the pre-trained models did not consistently give accurate classification results or perform well for the abnormal class. As such, this result supports the results and arguments presented in [7, 19, 29, 30] that classification models frequently favor the class with the highest weight.

In addition, we found that across all the classes, the average specificity is substantially higher than the average sensitivity. This revealed that while the models were good at accurately excluding the out-of-class samples from the relevant class, they struggled to correctly classify cells into the classes to which they belonged.

#### Evaluation of Models in Terms of the Class-by-Class Performance

**Fig. 11:**
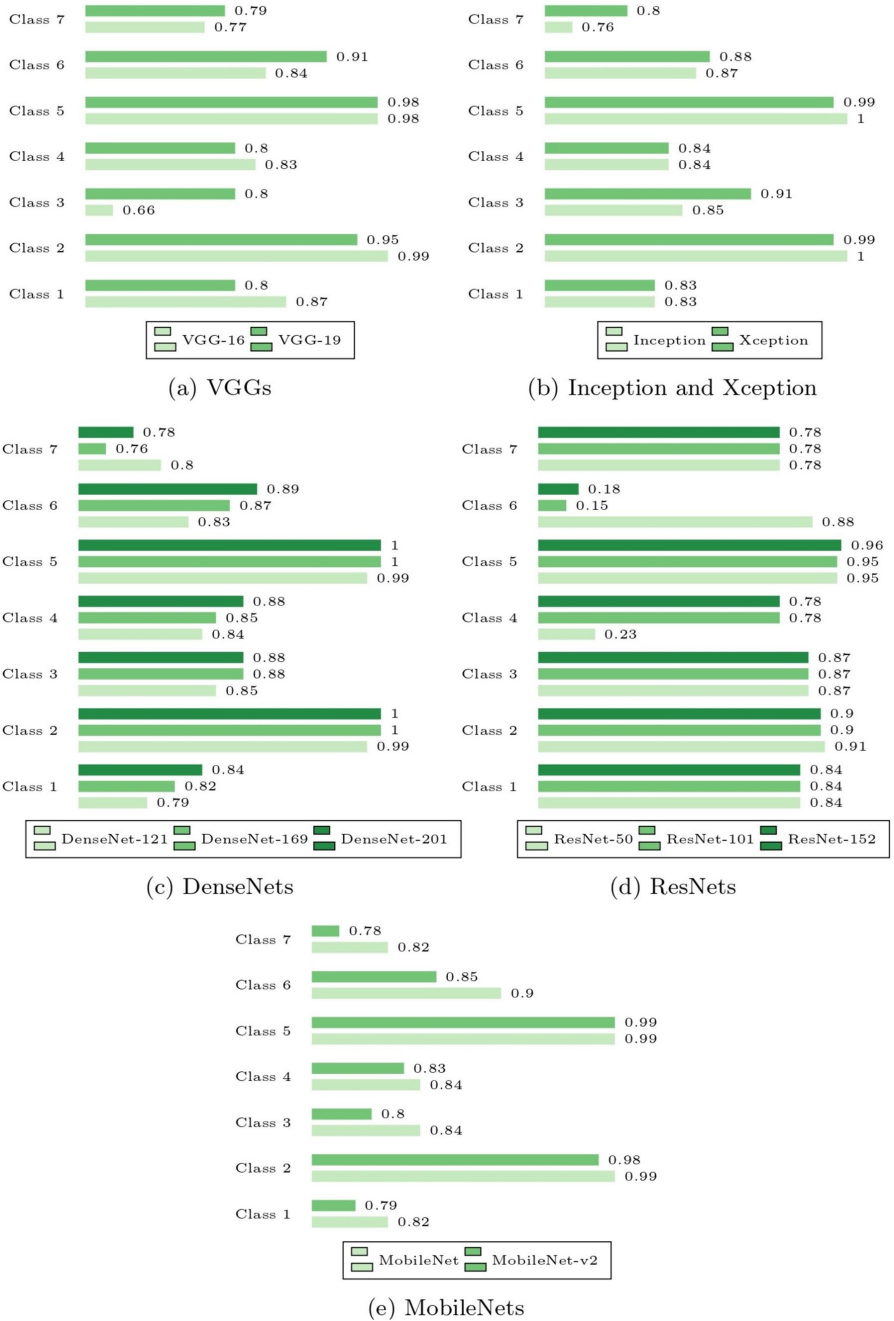
Class accuracy obtained by each model group

This section assessed the accuracy of multi-class classification in regard to the pre-trained CNN models’ performance. Figure 11 showed that no single model worked well for all classes simultaneously. For each class, the models performed differently. The following were observed:

- For the classification of Class 1, VGG-16 works best.
- For the classification of Class 2, Dense-Net-169, DenseNet-201, and Inception work best.
- For the classification of Class 3, Xception works best.
- For the classification of Class 4, DenseNet-201 work best.
- For the classification of Class 5, Dense-Net-169, DenseNet-201, and Inception work best.
- For the classification of Class 6, VGG-19 works best.
- For the classification of Class 7, MobileNet works best.

The findings mentioned above can imply that some models excel for particular cell attributes while others do not. The size of the samples for each class was also important. Additionally, the DenseNet-169, DenseNet-201, and Inception each obtained significant test accuracy with 1.0 for Class 2 and Class 5. Images from these two classes with a high degree of similarity are correctly classified by these models.

Additionally, all models, aside from the ResNets, perform best for a certain class. ResNets were not shown to be superior at classifying any particular classes. This suggests that the ResNets may preserve information from layers that provide little to no information or no information at all to the classification tasks, and instead, it appeared that the stored information made the models to perform worse as the count of layers increased.

In addition, the results for each type of CNN structure are analyzed and compared in this part. For all classes except Class 1, Class 2, and Class 4, VGG-19 outperformed VGG-16. Figure 11a shows that VGG-16 does not generally perform well for classes in the Normal class. It is concluded that deeper VGG networks perform better in abnormal cases whereas simpler VGG networks perform well in normal cases.

Xception is a variant of Inception that extends the intuition of Inception to its extreme. For all classes other than Class 2 and Class 5, Xception was found to be slightly superior to Inception as seen in Fig. 11b. This suggests that the notion of Xception, which employed various filter sizes and depthwise spatial correlation, is promising.

In Fig. 11c, for all classes except Class 7, classification accuracy improves as the DenseNets networks get deeper. However, its accuracy for Class 7 is variable. DenseNet-121 had the best accuracy, with DenseNet-201 and DenseNet-169 close behind. Notably, all three DenseNets had the same set of accuracy values for Class 2 and Class 5. As previously highlighted, Class 2 and Class 5 have very similar appearances. This could be one of the contributing factors to the observation and the fact that DenseNet dominates in these kinds of features.

In Fig. 11d, the performance of the smallest ResNet, RestNet-50, is the best of the three ResNets across all the classes, except for Class 4. Additionally, ResNet-50 performed remarkably well for Class 6 compared to the other classes, but ResNet-101 and ResNet-152 performed poorly. Moreover, as the network gets deeper, there was no improvement shown for Classes 1, 3, and 7. The observed results lead us to conclude that the ResNet structures fail to efficiently accomplish the multi-class classification problem in our case and that deeper ResNet networks were found to waste computational resources but do nothing to improve performance.

In Fig. 11e, the MobileNet was reported to be superior to the MobileNet-v2, but the performance for Class 5 was found to be on par for both models with an accuracy of 0.9878. Based on this finding, we presumed that MobileNet-v2 is inferior to MobileNet for multi-class classification on cervical cell images.

Overall, DenseNets were found to be superior to other pre-trained models for seven-class classification directly on the Herlev dataset without any pre-processing. As stated earlier, the DenseNets improved along with the increment of network layers, and the DenseNets-201 which is the deepest structure was found to be the best-performing model among the three DenseNets studied in this paper.

## Conclusion and Future Work

This study examined various pre-trained CNN models for detecting cervical cancer using publicly available datasets and built the groundwork for future agenda on automating cervical cancer detection and explored the effectiveness of DL models in multi-class classification problems. The key findings that addressed the various challenges in the literature are summarized below.

(i) Limited Data Size: In this study, all the pre-trained CNN models, except for ResNets, achieved accuracy levels higher than 80%. Additionally, all 13 CNN models were trained in less than 6 min. In the case of the small public Herlev dataset, transfer learning was shown to be a practical and relevant method for addressing time constraints and the scarcity of high-quality medical data.
(ii) Class Imbalance Problem: The models appeared to be particularly adept at classifying Class 2 and Class 5, and this was most likely because these two classes shared commonalities in appearance. Additionally, although some models were found to be superior at classifying a particular class of cells, none of the models were able to do it for all seven classes at once. Future studies should perform in-depth reviews of the strategies for addressing class imbalance in machine learning.
(iii) Reliant on Pre-processing Intervention: The deep CNN model that skipped the pre-segmentation and feature extraction stages with the best performance was found to be DenseNet-201, which trained in 2 min and 49 s and had the highest accuracy of 0.8702. Despite acquiring encouraging results with the models, it is possible to further improve their performance through hyperparameter tuning, additional testing, and ensemble techniques.
(iv) Generalizability of Models: To the best of our knowledge, this study is the first in comparing 13 CNN models and extensively assesses each model’s performance in cervical cancer classification on a class-by-class basis.

## Data Availability

This study uses open-source cervical cancer datasets from the Herlev database that can be accessed via https://mde-lab.aegean.gr/downloads, an open-source online data repository hosted at MDE-Lab (https://mde-lab.aegean.gr/).
